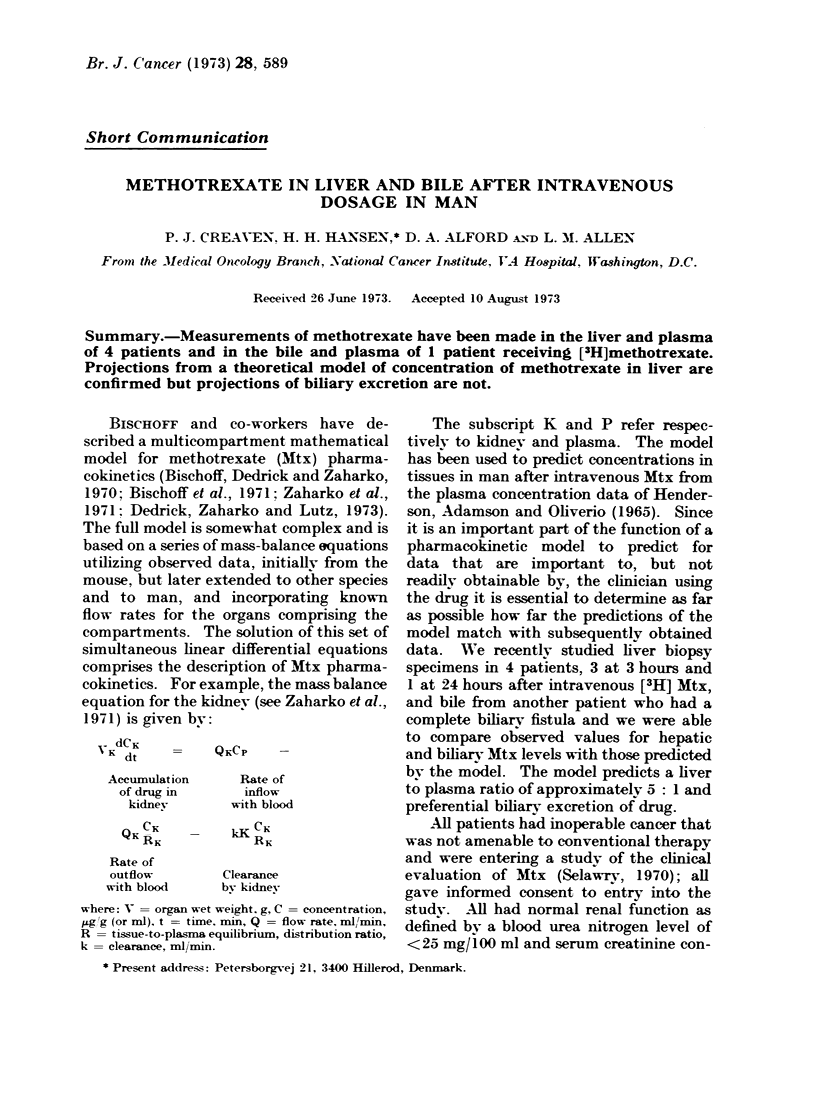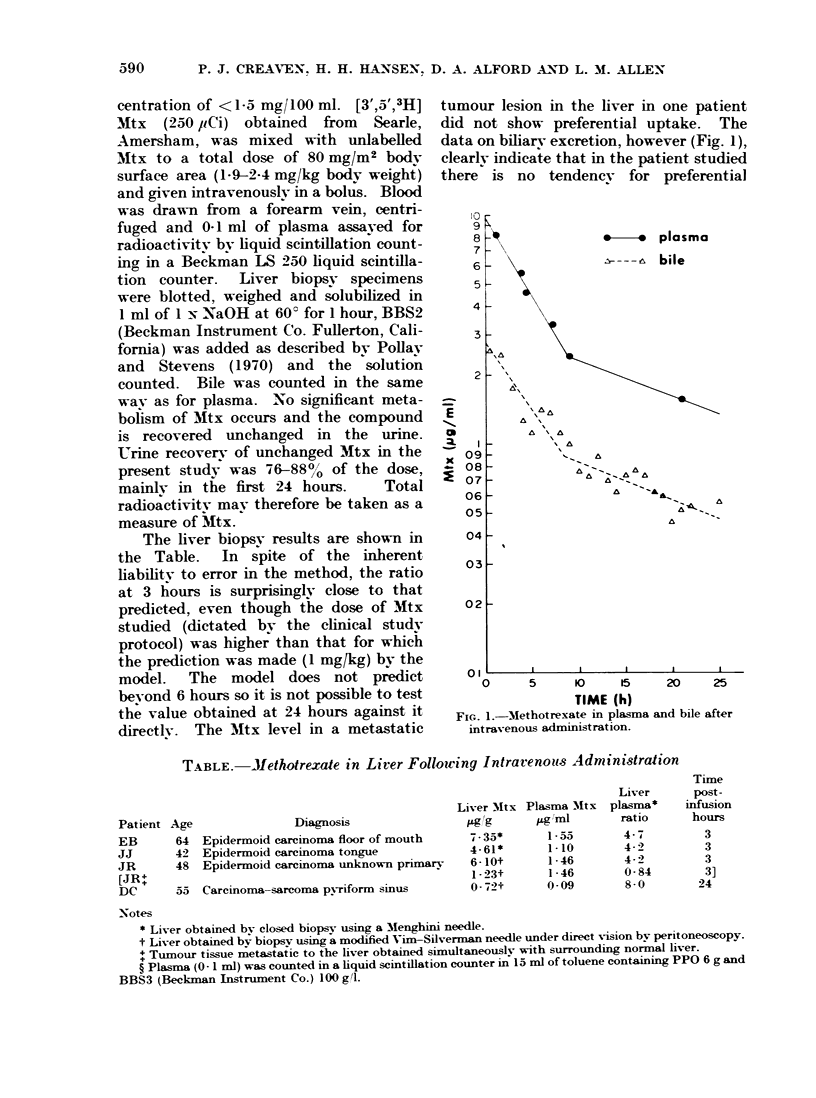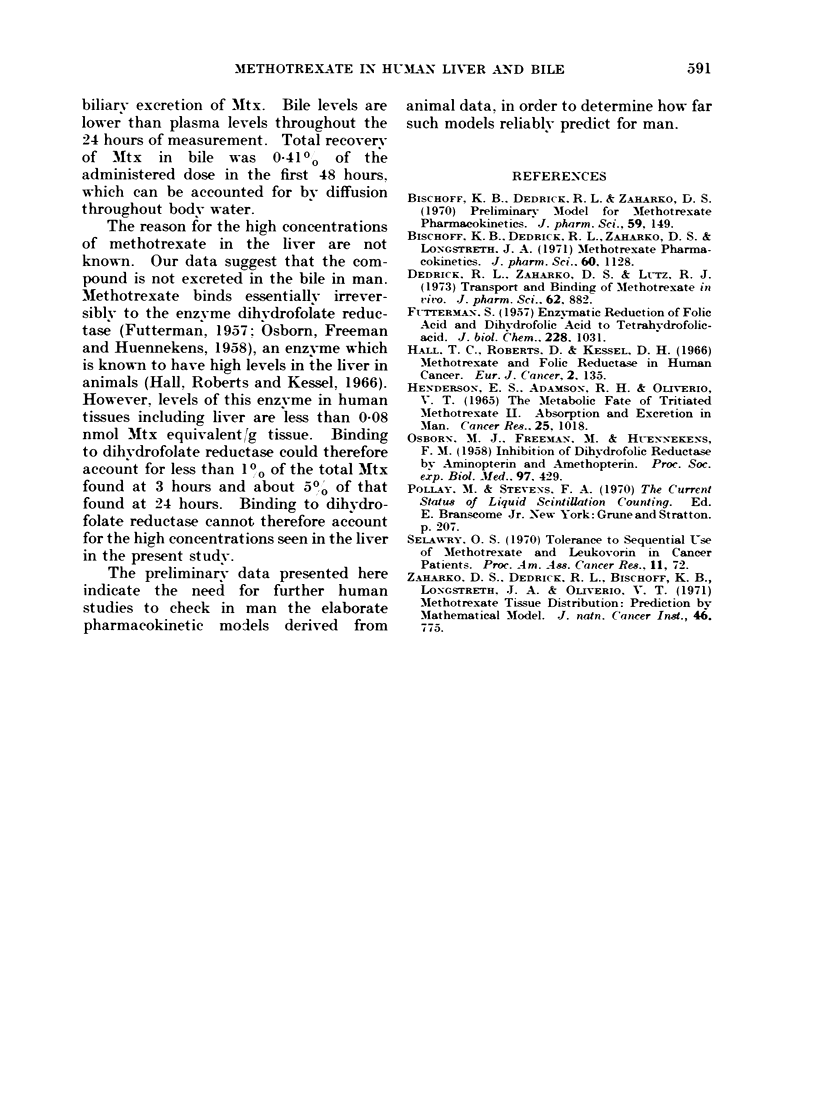# Methotrexate in Liver and Bile after Intravenous Dosage in Man

**DOI:** 10.1038/bjc.1973.190

**Published:** 1973-12

**Authors:** P. J. Creaven, H. H. Hansen, D. A. Alford, L. M. Allen

## Abstract

Measurements of methotrexate have been made in the liver and plasma of 4 patients and in the bile and plasma of 1 patient receiving [^3^H]methotrexate. Projections from a theoretical model of concentration of methotrexate in liver are confirmed but projections of biliary excretion are not.


*Br. J. Cancer* (1973) **28**, 589

***Short Communication***

### METHOTREXATE IN LIVER AND BILE AFTER INTRAVENOUS DOSAGE IN MAN

P. J. CREAVEN, H. H. HANSEN,* D. A. ALFORD AND L. M. ALLEN

*From the Medical Oncology Branch, National Cancer Institute, VA Hospital, Washington, D.C.*

Received 26 June 1973. Accepted 10 August 1973

**Summary.—Measurements of methotrexate have been made in the liver and plasma of 4 patients and in the bile and plasma of 1 patient receiving [$^3$H]methotrexate. Projections from a theoretical model of concentration of methotrexate in liver are confirmed but projections of biliary excretion are not.**

BISCHOFF and co-workers have described a multicompartment mathematical model for methotrexate (Mtx) pharmacokinetics (Bischoff, Dedrick and Zaharko, 1970; Bischoff *et al.*, 1971; Zaharko *et al.*, 1971; Dedrick, Zaharko and Lutz, 1973). The full model is somewhat complex and is based on a series of mass-balance equations utilizing observed data, initially from the mouse, but later extended to other species and to man, and incorporating known flow rates for the organs comprising the compartments. The solution of this set of simultaneous linear differential equations comprises the description of Mtx pharmacokinetics. For example, the mass balance equation for the kidney (see Zaharko *et al.*, 1971) is given by:

$$V_K \frac{dC_K}{dt} = Q_K C_P - Q_K \frac{C_K}{R_K} - kK \frac{C_K}{R_K}$$

| $V_K \frac{dC_K}{dt}$ | $Q_K C_P$ | $Q_K \frac{C_K}{R_K}$ | $kK \frac{C_K}{R_K}$ |
|---|---|---|---|
| Accumulation of drug in kidney | Rate of inflow with blood | Rate of outflow with blood | Clearance by kidney |

where: $V$ = organ wet weight, g, $C$ = concentration, μg/g (or ml), $t$ = time, min, $Q$ = flow rate, ml/min, $R$ = tissue-to-plasma equilibrium, distribution ratio, $k$ = clearance, ml/min.

The subscript K and P refer respectively to kidney and plasma. The model has been used to predict concentrations in tissues in man after intravenous Mtx from the plasma concentration data of Henderson, Adamson and Oliverio (1965). Since it is an important part of the function of a pharmacokinetic model to predict for data that are important to, but not readily obtainable by, the clinician using the drug it is essential to determine as far as possible how far the predictions of the model match with subsequently obtained data. We recently studied liver biopsy specimens in 4 patients, 3 at 3 hours and 1 at 24 hours after intravenous [$^3$H] Mtx, and bile from another patient who had a complete biliary fistula and we were able to compare observed values for hepatic and biliary Mtx levels with those predicted by the model. The model predicts a liver to plasma ratio of approximately 5 : 1 and preferential biliary excretion of drug.

All patients had inoperable cancer that was not amenable to conventional therapy and were entering a study of the clinical evaluation of Mtx (Selawry, 1970); all gave informed consent to entry into the study. All had normal renal function as defined by a blood urea nitrogen level of $<25$ mg/100 ml and serum creatinine con-

* Present address: Petersborgvej 21, 3400 Hillerod, Denmark.

centration of <1·5 mg/100 ml. [3′,5′,$^3$H] Mtx (250 $\mu$Ci) obtained from Searle, Amersham, was mixed with unlabelled Mtx to a total dose of 80 mg/m$^2$ body surface area (1·9–2·4 mg/kg body weight) and given intravenously in a bolus. Blood was drawn from a forearm vein, centrifuged and 0·1 ml of plasma assayed for radioactivity by liquid scintillation counting in a Beckman LS 250 liquid scintillation counter. Liver biopsy specimens were blotted, weighed and solubilized in 1 ml of 1 N NaOH at 60° for 1 hour, BBS2 (Beckman Instrument Co. Fullerton, California) was added as described by Pollay and Stevens (1970) and the solution counted. Bile was counted in the same way as for plasma. No significant metabolism of Mtx occurs and the compound is recovered unchanged in the urine. Urine recovery of unchanged Mtx in the present study was 76–88% of the dose, mainly in the first 24 hours. Total radioactivity may therefore be taken as a measure of Mtx.

The liver biopsy results are shown in the Table. In spite of the inherent liability to error in the method, the ratio at 3 hours is surprisingly close to that predicted, even though the dose of Mtx studied (dictated by the clinical study protocol) was higher than that for which the prediction was made (1 mg/kg) by the model. The model does not predict beyond 6 hours so it is not possible to test the value obtained at 24 hours against it directly. The Mtx level in a metastatic tumour lesion in the liver in one patient did not show preferential uptake. The data on biliary excretion, however (Fig. 1), clearly indicate that in the patient studied there is no tendency for preferential

FIG. 1.—Methotrexate in plasma and bile after intravenous administration.

TABLE.—*Methotrexate in Liver Following Intravenous Administration*

| Patient | Age | Diagnosis | Liver Mtx µg/g | Plasma Mtx µg/ml | Liver plasma* ratio | Time post-infusion hours |
|---|---|---|---|---|---|---|
| EB | 64 | Epidermoid carcinoma floor of mouth | 7·35* | 1·55 | 4·7 | 3 |
| JJ | 42 | Epidermoid carcinoma tongue | 4·61* | 1·10 | 4·2 | 3 |
| JR | 48 | Epidermoid carcinoma unknown primary | 6·10† | 1·46 | 4·2 | 3 |
| [JR‡ | | | 1·23† | 1·46 | 0·84 | 3] |
| DC | 55 | Carcinoma–sarcoma pyriform sinus | 0·72† | 0·09 | 8·0 | 24 |

Notes
* Liver obtained by closed biopsy using a Menghini needle.
† Liver obtained by biopsy using a modified Vim–Silverman needle under direct vision by peritoneoscopy.
‡ Tumour tissue metastatic to the liver obtained simultaneously with surrounding normal liver.
§ Plasma (0·1 ml) was counted in a liquid scintillation counter in 15 ml of toluene containing PPO 6 g and BBS3 (Beckman Instrument Co.) 100 g/l.

biliary excretion of Mtx. Bile levels are lower than plasma levels throughout the 24 hours of measurement. Total recovery of Mtx in bile was 0·41% of the administered dose in the first 48 hours, which can be accounted for by diffusion throughout body water.

The reason for the high concentrations of methotrexate in the liver are not known. Our data suggest that the compound is not excreted in the bile in man. Methotrexate binds essentially irreversibly to the enzyme dihydrofolate reductase (Futterman, 1957; Osborn, Freeman and Huennekens, 1958), an enzyme which is known to have high levels in the liver in animals (Hall, Roberts and Kessel, 1966). However, levels of this enzyme in human tissues including liver are less than 0·08 nmol Mtx equivalent/g tissue. Binding to dihydrofolate reductase could therefore account for less than 1% of the total Mtx found at 3 hours and about 5% of that found at 24 hours. Binding to dihydrofolate reductase cannot therefore account for the high concentrations seen in the liver in the present study.

The preliminary data presented here indicate the need for further human studies to check in man the elaborate pharmacokinetic models derived from animal data, in order to determine how far such models reliably predict for man.

### REFERENCES

Bischoff, K. B., Dedrick, R. L. & Zaharko, D. S. (1970) Preliminary Model for Methotrexate Pharmacokinetics. *J. pharm. Sci.*, **59**, 149.

Bischoff, K. B., Dedrick, R. L., Zaharko, D. S. & Longstreth, J. A. (1971) Methotrexate Pharmacokinetics. *J. pharm. Sci.*, **60**, 1128.

Dedrick, R. L., Zaharko, D. S. & Lutz, R. J. (1973) Transport and Binding of Methotrexate *in vivo*. *J. pharm. Sci.*, **62**, 882.

Futterman, S. (1957) Enzymatic Reduction of Folic Acid and Dihydrofolic Acid to Tetrahydrofolic-acid. *J. biol. Chem.*, **228**, 1031.

Hall, T. C., Roberts, D. & Kessel, D. H. (1966) Methotrexate and Folic Reductase in Human Cancer. *Eur. J. Cancer*, **2**, 135.

Henderson, E. S., Adamson, R. H. & Oliverio, V. T. (1965) The Metabolic Fate of Tritiated Methotrexate II. Absorption and Excretion in Man. *Cancer Res.*, **25**, 1018.

Osborn, M. J., Freeman, M. & Huennekens, F. M. (1958) Inhibition of Dihydrofolic Reductase by Aminopterin and Amethopterin. *Proc. Soc. exp. Biol. Med.*, **97**, 429.

Pollay, M. & Stevens, F. A. (1970) *The Current Status of Liquid Scintillation Counting.* Ed. E. Branscome Jr. New York: Grune and Stratton. p. 207.

Selawry, O. S. (1970) Tolerance to Sequential Use of Methotrexate and Leukovorin in Cancer Patients. *Proc. Am. Ass. Cancer Res.*, **11**, 72.

Zaharko, D. S., Dedrick, R. L., Bischoff, K. B., Longstreth, J. A. & Oliverio, V. T. (1971) Methotrexate Tissue Distribution: Prediction by Mathematical Model. *J. natn. Cancer Inst.*, **46**, 775.